# Supplementary material for: Phenotypic Characterization of Circulating Tumor Cells Isolated from Non-Small and Small Cell Lung Cancer Patients
Source: Cancers (Basel). 2022 Dec 28;15(1):171. doi: 10.3390/cancers15010171 (PMC9818148; doi:10.3390/cancers15010171)
Supplement: Supplementary file 1 [file cancers-15-00171-s001.zip › cancers-2088493-supplementary.pdf]

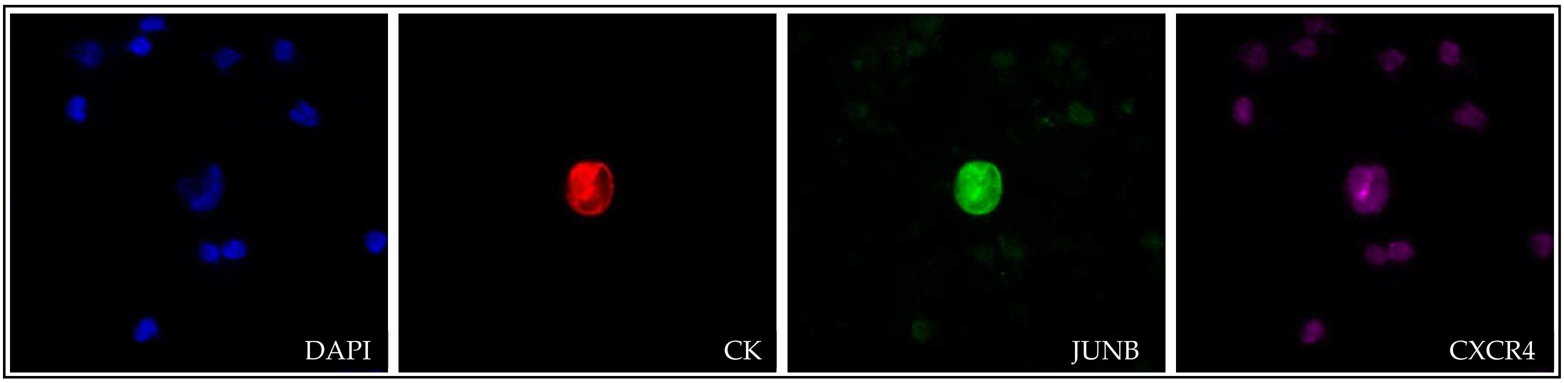

Figure S1: Cytokeratin (red), JUNB (green) and CXCR4 (purple) expression in a H1299 cell spiked in peripheral blood mononuclear cells (PBMCs) isolated from a healthy donor (VyCAP imaging system). Magnification 20X.

Table S1: Number of CTCs per phenotype and per patient in NSCLC cases.

NSCLC					
**Patient**	**CK+CXCR4+JUNB+**	**CK+CXCR4-JUNB+**	**CK+CXCR4+JUNB-**	**CK+CXCR4-JUNB-**	**total phenotypes per patient**
1	0	0	0	0	0
2	0	0	0	0	0
3	0	0	0	0	0
*4	1	0	0	0	1
*5	0	2	0	0	1
*6	4	0	0	0	1
*7	0	1	0	0	1
8	0	0	0	0	0
9	0	0	0	0	0
10	0	0	0	0	0
*11	3	0	0	0	1
*12	0	1	0	0	1
13	0	2	0	1	2
14	0	3	0	6	2
15	0	4	0	1	2
*16	3	0	0	0	1
*17	0	0	0	3	1
18	1	0	0	1	2
19	0	0	0	0	0
20	0	0	0	0	0
21	0	0	0	0	0
*22	3	0	0	0	1
23	0	0	0	0	0
24	0	0	0	0	0
*25	1	0	0	0	1
26	0	0	0	0	0
27	0	0	0	0	0
28	1	2	0	1	3
29	0	0	0	0	0
*30	0	0	1	0	1

Table S2: Number of CTCs per phenotype and per patient in SCLC cases.

SCLC					
Patient	CK+JUNB+CXCR4+	CK+JUNB+CXCR4-	CK+JUNB-CXCR4+	CK+JUNB-CXCR4-	total phenotypes per patient
1	0	1	1	3	3
2	2	17	0	18	3
3	1	22	1	4	4
4	4	2	1	23	4
5	0	3	0	1	2
6	5	0	1	3	3
7	2	2	0	20	3
8	1	0	2	2	3
9	5	0	0	6	2
*10	2	0	0	0	1
11	0	0	2	105	2
12	4	6	2	31	4
13	0	1	2	2	3
14	2	0	1	2	3
15	1	2	12	33	4
16	2	3	1	60	4
*17	0	1	0	0	1
18	1	0	1	6	3
19	0	0	0	0	0
20	0	0	0	0	0
21	2	6	3	33	4
22	0	0	0	0	0
23	0	0	1	1	2
24	0	1	0	1	2
*25	0	2	0	0	1
26	0	0	0	0	0
27	0	0	0	0	0
28	0	0	0	0	0
29	0	2	0	1	2
30	3	6	1	5	4
31	11	5	14	6	4
32	1	0	1	0	2
33	2	1	2	1	4
34	4	7	6	10	4
35	9	5	2	2	4

36	2	5	1	3	4
37	4	5	1	0	3
